# Antioxidant properties and potential mechanisms of hydrolyzed proteins and peptides from cereals

**DOI:** 10.1016/j.heliyon.2019.e01538

**Published:** 2019-04-29

**Authors:** Ramak Esfandi, Mallory E. Walters, Apollinaire Tsopmo

**Affiliations:** a Food Science and Nutrition Program, Department of Chemistry, Carleton Uinversity, 1125 Colonel By Drive, K1S 5B6, Ottawa, ON, Canada; b Institute of Biochemistry, Carleton University, 1125 Colonel By Drive, K1S 5B6, Ottawa, ON, Canada

**Keywords:** Nutrition, Food science

## Abstract

Cereals like wheat, rice, corn, barley, rye, oat, and millet are staple foods in many regions around the world and contribute to more than half of human energy requirements. Scientific publications contain evidence showing that apart from energy, the regular consumption of whole grains is useful for the prevention of many chronic diseases associated with oxidative stress. Biological activities have mostly been attributed to the presence of glucans and polyphenols. In recent years however, food proteins have been investigated as sources of peptides that can exert biological functions, promote health and prevent oxidative stress. This review focuses on the role of hydrolyzed proteins and peptides with antioxidant properties in various models and their mechanisms which include hydrogen or electron transfer, metal chelating, and regulation of enzymes involved in the oxidation-reduction process.

## 1 Introduction

Cereals are major sources of energy for populations around the world. Taxonomic classification places them in the Gramineae or Poaceae family which is divided into seven major subfamilies of grasses, *Bambusoideae*, *Oryzoideae*, *Pooideae*, *Panicoideae*, *Arundinoideae*, *Chloridoideae*, and *Centothecoideae*, 40 tribes and about 600–750 genera [1]. Wheat, barley, and rye belong to the genus *Triticale*, oats to *Aveneae*, rice to *Oryzeae*, corn and sorghum to *Andropogoneae*, and millet to *Panicease* [2]. Wheat and rice are the most important cereals with regard to human nutrition, and account for up 55% of the total cereal production [3]. Worldwide, cereals contribute to about half of the human energy requirement and have been grown as staple foods for human consumption and feeds for livestock since the beginning of civilization [4]. The major cereal grains consumed in descending order are wheat, rice, corn (or maize), barley, oats, rye, millet, and sorghum. These grains provide carbohydrates, proteins, B-vitamins, and minerals for a major portion of the world's population [3]. Cereals are also rich in secondary metabolites (*i.e*. bioactive compounds) that are important in health promotion. The majority of bioactive compounds are located within whole grains, bran or germ rich milling fractions. Many studies therefore have been performed to investigate the beneficial effect of whole grains or milling fractions for the general well-being, and for the reduction of oxidative stress and associated disease conditions such as cardiovascular diseases, diabetes, obesity, and cancer [5, 6, 7, 8].

The beneficial effects of cereals have mostly been attributed to the presence of fibers (e.g. glucans), a group of polysaccharides that are resistant to digestive enzymes in the gastro-intestinal tract, meanwhile their health promoting effects go beyond fibers. In women for example, the health effects of whole grains on heart disease remained even after controlling the intake of fibers [9]. While in men, the bran had a better protection against heart disease than whole grains [10], likely because of both fibres and phytochemicals. The benefit of whole grains, therefore, comes from more than one group of compounds that might act in additive or synergistic manners. In recent years, peptides and proteins present in foods have gradually been recognized as positive contributors to human health and as such, there has been an increase of studies that focus on the identification and physiological properties of hydrolyzed dietary proteins or purified peptides [11, 12]. Peptides or their mixtures used in many investigations are generally produced by chemical hydrolysis, enzymatic hydrolysis, or microbial fermentation of protein concentrates or isolates [13]. In the food industry, the method of choice is enzymatic hydrolysis because of the absence of toxic chemical residues. Proteolytic enzymes can come from microbes, plants, or animals and cleave proteins only at certain peptide bonds because of differences in their specificities [14]. Other factors such as time, temperature, pH, and origin of proteins have an influence on the sequence of resulting peptides and their biological properties. After hydrolysis, ultrafiltration and chromatographic procedures are commonly used to obtain completely pure peptides or peptide fractions of different molecular weights, ionisation states or hydrophobicity. The majority of bioactive peptides in the literature are short (less than twenty amino acid residues), and their activities are affected by both amino acid compositions and sequences [12, 15]. Biological functions such as antioxidant, antihypertensive, and antitumor activities have been reported for cereal protein hydrolysates or derived peptides [16, 17]. This review focuses on those that possess anti-oxidative properties in foods and biological systems.

## 2 Main text

### 2.1 Oxidation and oxidative stress

Oxidative stress occurs as a result of an imbalance between the amount of oxidants produced and the antioxidant defense mechanism. There are endogenous and exogenous oxidants. Two groups of oxidants commonly referred to as reactive oxygen species (ROS) and reactive nitrogen species (RNS) are the main contributors. However, chlorine, bromine, and sulfide reactive species are contributors as well to the alteration of the redox balance. Most oxidants are unstable and short-lived chemical species generated under various conditions in foods and physiological conditions.

#### 2.1.1 Sources of oxidants in food and biological systems

Processes that generate oxidants in food include the length of storage and temperature, heat treatment, the amount of available oxygen, the presence of transition metals, or oxidative enzymes. Thermal oxidation of lipids, for example, form unstable primary oxidation products (i.e. hydroperoxides) through initial proton removal and oxygen consumption [12]. The hydroperoxides are then decomposed into secondary oxidation products such as aldehydes and ketones [18]. Phytochemicals (i.e. secondary metabolites) such as carotenoids and polyphenols have antioxidant properties, meanwhile, they also possess pro-oxidant function due to the formation of peroxides [12, 19]. Phenoxyl radicals, often quinones and semiquinones, are formed via auto-oxidation reactions of phenol moieties in the presence of radicals, hydrogen peroxide, and divalent metal ions [19, 20]. Hydroxyl radicals generated from the reaction of hydrogen peroxide with metals such as ferrous ions are also present in foods [21]. Light accelerates oxidation, especially in the presence of photosensitizers such as chlorophylls and riboflavin, which become excited upon absorption of energy [22]. Once excited, chlorophyll or riboflavin reacts with triplet oxygen to produce singlet oxygen by energy transfer and return to their ground singlet state. The excited oxygen can diffuse [23] and oxidize electron-rich compounds such as unsaturated lipids, some amino acids and peptides.

In biological systems, the primary cellular sources of ROS and RNS are the mitochondria where oxidative phosphorylation occurs. The mitochondria electron transport chain is composed of four multi-protein complexes I-IV and comprises a series of electron carriers such as ubiquinone, cytochromes, flavoproteins, and iron-sulfur proteins. The domains are arranged spatially according to their redox potentials, which vary from −0.320 to +0.380 V [24]. In complex I, electrons derived from metabolic reducing equivalents of NADH are transferred via multiple redox cofactors to the first mobile electron carrier, oxidized coenzyme Q (CoQ). The energy released from the reaction is then captured via ejection of four protons from the mitochondrial matrix into the intermembrane space [25, 26]. Electrons are also transferred to CoQ from flavoproteins, glycerol 3-phosphate, and succinate dehydrogenase (complex II). Reduced CoQ then transfers electrons to complex III for ultimate collection by cytochrome C, which is present in the inter-mitochondrial space. The energy liberated is captured via a proton pumping mechanism. The last step is the reduction of molecular oxygen to water in four one-electron steps in complex IV. It is estimated that 0.2–2% of the total oxygen consumption is reduced to superoxide anion radicals during respiration in mitochondria [27]. This process leads to the production of other ROS species specifically in complexes I and III (about 90% total production). Organelles such as the endoplasmic reticulum and nuclear membranes also contain electron transport chains capable of donating electrons to molecular oxygen, thereby generating superoxide anion radicals and subsequent oxidants [28]. During their catalytic processes, various enzymes that include xanthine oxidase, cytochrome P450 mono-oxygenase, nitric oxide synthases, lipoxygenases, cyclo-oxygenase, and NADPH oxidase also generate ROS and RNS [26, 28]. The consumption of oxidized foods and the exposure to radiation of environmental toxins (metals, chlorinated compounds) are in vivo sources of oxidants.

#### 2.1.2 Oxidant species and deleterious effects

Common ROS and RNS include free radicals like superoxide anion (O_2_^·−^), hydroxyl (HO^·^), nitric oxide (NO^·^), nitrogen dioxide (NO_2_^·^), and peroxyl (ROO^·^), as well as non-radical species like singlet oxygen (^1^O_2_), hydrogen peroxide (H_2_O_2_), ozone (O_3_), hypochlorous acid (HOCl), nitrous acid (HNO_2_), peroxynitrite (ONOO^−^), dinitrogen trioxide (N_2_O_3_), and hydroperoxide (ROOH) [5, 29]. Oxidation of unsaturated fatty acids in foods by ^1^O_2_, O_2_^·−^, or HO^·^ through successive reactions generate aldehyde compounds that are responsible for off-flavor characteristics of many food products [12, 23]. Phenoxyl radicals as well as HO^·^ and ROO^·^ can decrease the nutritional value of proteins by reacting with nucleophilic groups, such as the side-chain amine group of lysine or the sulfhydryl group of cysteine in free amino acids, peptides, and proteins [12, 21, 30].

ROS released by mitochondria are believed to play an important role in conditions associated with oxidative stress such as the aging process, neurodegeneration (e.g. Parkinson's disease), and atherosclerosis [24, 31]. This is because ROS and RNS induce damage to various biomolecules including DNA strand breaks, base and nucleotide modifications, particularly in sequences containing high guanosine [32, 33]. The oxidation of amino acid residues can lead to the formation of protein aggregates through cross-linking, loss of enzyme activity, poor metabolic pathways, or cell death [33]. Carbohydrates can also be oxidized and lead to accumulation of advanced glycation end-products which can further react with other molecules, thereby contributing for example to increase vascular permeability or low-density lipoprotein (LDL) oxidation [34]. LDL oxidation is a contributor to many diseases such as cardiovascular diseases, arthritis, dementia and metabolic syndrome [35]. Oxidation of lipids to hydroperoxides can disrupt cell membrane integrity and can also damage proteins [5].

#### 2.1.3 Positive role of oxidant molecules

There is no known beneficial role of oxidants in foods, but they exist in biological systems. At low or moderate levels, ROS and RNS play important role in the maturation process of cellular structures and maintenance of the host defense system [29]. During inflammation and infection, phagocytes (neutrophils, macrophages, monocytes) release free radicals to destroy invading pathogenic microbes as part of the body's defense mechanism [29, 36]. Although the excess concentration of oxidants is detrimental to organisms, very low levels can be detrimental as well. It has been found, for example, that patients with granulomatous disease, a condition that results in defective membrane-bound NADPH oxidase, suffer from multiple and persistent infections because of their inability to produce (O_2_^·^ˉ) radicals [37]. Other beneficial effects of ROS and RNS involve their physiological roles in the function of cellular signaling systems like the sensing of OxyR and SoxR transcription factors which are involved in DNA repair [38, 39].

### 2.2 Antioxidative properties of protein hydrolysates and peptides from cereals

Proteins represent about 6–15% of cereals grains. In general, there are two main groups of proteins: prolamins and globulins [40]. In wheat, rye, barley, and corn prolamins constitute 30–50% of total proteins and are called gliadins, secalins, hordeins and zeins, respectively [41, 42]. In oats and rice, the presence of prolamins is minor and they account for 5–15 % of proteins [40]. The nutritional quality of cereal proteins is low due to limitations in essential amino acids, mainly lysine. This is more pronounced in those cereals with high prolamin contents. In addition, these proteins possess limited functionality due to low water solubility and their hydrolysis is viewed as a way to improve not only nutritional values, but also to release bioactive peptides.

#### 2.2.1 Summary of the production of hydrolyzed cereal proteins

The initial step is the extraction of proteins which can be achieved by solubilisation of cereal flours in solution of salts such as KCl and NaCl, or at alkaline conditions (pH 9–10) [43, 44]. The alkali method is the most commonly used procedure, meanwhile due to high content sugars in cereals, pre-treatments with polysaccharide degrading enzymes (e.g. cellulase, amylase, viscozyme) have been used to enhance the solubilisation of proteins which are then precipitated at isoelectric points [45, 46]. The next step involves the cleavage of bonds in the extracted proteins using proteases or by fermentation with microorganisms. These steps lead to the production of protein hydrolysates which has been found to possess various activities. Fractionation of protein hydrolysates are necessary in many cases to concentrate the activity into specifies fractions or to facilitate the identification of peptides. Common methods of fractionation are based on size (membrane filtration, gel chromatography), charge (ion-exchange chromatography), and hydrophobicity (reverse phase chromatography) [47, 48, 49]. The identification of peptides in the hydrolysates and fractions is often achieved using tandem mass spectrometric and bioinformatic techniques as reviewed in a recent paper [50].

#### 2.2.2 Radical scavenging activities

One of the well known and commonly investigated properties of antioxidant molecules is their ability to scavenge radicals. It is, therefore, not surprising that data on the quenching of radicals by hydrolyzed cereal proteins exist in the literature.

##### 2.2.2.1 Activities of protein hydrolysates

Wheat germ proteins hydrolysed with Proleather FG-F, a protease from Bacillus subtilis, scavenged 81% DPPH (1.6 mg/mL) and 75% O_2_^·−^ (0.6 mg/mL) radicals [51]. In other works, alcalase hydrolysed wheat germ proteins quenched DPPH, O_2_^·−^, and HO^·^ radicals with EC_50_ values of 1.3, 0.4 and 0.1 mg/mL, respectively [52]. In comparison, the EC_50_ values for wheat proteins fermented with Bacillus Subtilis B1 were 3.2 mg/mL, 6.0 mg/mL and 7.5 mg/mL, respectively [53]. It appears that alcalase was more effective in releasing O_2_^·−^ and HO^·^ radical scavenging peptides from wheat germ while Proleather FG-F was better for releasing peptides that quenched DPPH radicals. For rice dreg proteins, protamex hydrolysates had better DPPH activity (EC_50_ 8.7 mg/mL) than those from alcalase, neutrase, flavourzyme, and trypsin treatments (EC_50_ 9.9–14.0 mg/mL) [54]. Meanwhile, rice endosperm proteins hydrolysed with neutrase possessed DPPH (EC_50_ 0.05 mg/mL) and hydroxyl radical (EC_50_ 2.0 mg/mL) scavenging activities, as well as a weak superoxide anion radical scavenging activity [55]. In a related study, rice bran protein hydrolysate prepared with a mixture of papain and flavourzyme displayed the highest DPPH activity (IC_50_ 6.8 mg/mL) compared to values of hydrolysates prepared with a combination of two proteases comprising papain, flavourzyme, neutrase, protamex, or trypsin [56]. Wang et al. [57] reported IC_50_ of 1.57 mg/mL for DPPH radical scavenging activity of a trypsin digest. Treatment of rice proteins with neutrase, alcalase, and flavourzyme produced hydrolysates with ABTS^·^ scavenging activities (89–151 μg ascorbic acid (AA) equivalent/mL) relative to treatments with microbial cell extracts (220–280 μg AA eq/mL) [58]. Based on these studies, rice dreg hydrolysates have much lower scavenging activities than those of brans. They are also lower relative to wheat germ or gluten hydrolysates.

Corn gluten proteins treated with various proteases possess antioxidant activities against HO^·^ (E_C50_ 0.8–7.5 mg/mL), O_2_^·−^ (EC_50_ 12.5–12.8 mg/mL), and DPPH (EC_50_ 1.0–1.26 mg/mL) radicals [59, 60]. Hydrolyzed corn proteins and fractions also scavenged ROO^·^ radicals with values of 65.6–191.4 μM TE/g [61]. Alcalase hydrolyzed Hordein proteins and its fractions had DPPH radical scavenging activities with EC_50_ values from 0.5 to 3.8 mg/mL or 48–58%, at 0.5 mg/mL, and quenched O_2_^·−^ by up to 40% at 0.5–1.0 mg/mL [62, 63]. In a related work O_2_^·−^ and HO^·^ scavenging activities of barley gluten alcalase hydrolysates were stronger than those of flavourzyme digests, however, DPPH activity was lower [64]. Hydrolyzed oat bran proteins had ROO^·^ radical scavenging activities of 343–608 μM TE/g and inhibited the formation of O_2_^·−^ by 20–36% and HO^·^ by 10.2–14.1%, depending on the hydrolysis time and the concentration of pepsin [49]. Treatment of oat bran proteins with protamex (various concentrations and time) provide similar ROO^·^ (408–712 μM TE/g) activities, but lower HO^·^ (2.4–11.2%) and higher O_2_^·−^ (24–58%) scavenging activities [65]. Alcalase and trypsin hydrolysate from whole oat flour ROO^·^ activities were 269 and 434 μM TE/g, respectively while DPPH radicals quenching were 20–35% at 1 mg/mL [43].

Fractionation of hydrolysed proteins can concentrate activities within certain fractions. Wheat pepsin (<3 kDa) and papain (<5 kDa) gluten hydrolysates had higher DPPH, HO^·^, and O_2_^·−^ activities relative to fractions with higher molecular weights [66, 67, 68]. The influence of chemical modifications is illustrated by the increased DPPH and HO^·^ activities after conjugation of wheat gluten hydrolysates (alcalase and flavourzyme) with glucosamine [69], and a decreased ROO^·^ in scavenging activity of pepsin and pancreatin hydrolysates upon deamidation with citric acid [70]. Polysaccharide degrading enzymes have been used to enhance both extraction yield of proteins and radical scavenging properties of hydrolysates [44]. Ultrasonic-assisted enzymolysis and pulsed electric field technology increased DPPH or HO^·^ activities of corn protein hydrolysates [71, 72]. Other strategies to enhance the activity include the choice of protease (however, there appears to be no ideal one because of the influence of concentration), temperature, and duration of hydrolysis. In addition, the alkaline pH will affect protein solubility, extraction yield and composition, and the properties of subsequently generated hydrolysates.

##### 2.2.2.2 Activities of peptides

The number of studies performed on pure peptides is limited. This is likely because it is labor intensive to fraction and isolate individual peptides using available chromatography and membrane technologies. Meanwhile, many peptides have been identified in hydrolysates or fractions with antioxidant activities but only a few have been tested (Table 1).

**Table 1:** Sequences of sources of cereal antioxidant peptides.

Food source	Peptide sequence	Antioxidant assay	Reference
Oat	GLVYIL YHNAP FNDRLRQGQLL GQTV GQTVFNDRLRQGQLL YHNAPGLVYIL DVNNNANQLEPR	ORAC (peroxyl radical scavenging), hepatic HepG2 cells, antioxidant enzymes	[73]
Wheat	VLPPQQQY TVTSLDLPVLRW VTSLDLPVLRW STTTGHLIYK TVVPPKGGSFYPGETTP FVPY	ABTS, DPPH, O_2_^·ˉ^ and HO^·^ scavenging	[74]
	RVF	Human neuroblastoma SH-SY5Y cells, enhance total antioxidant capacity	[108]
Rice residue proteins	RPNYTDA TSQLLSDQ TRTGDPFF NFHPQ	DPPH, ABTS, and FRAP reducing assay	[75]
Rice endosperm	FRDEHKK	Linoleic acid oxidation inhibition, intracellular oxidant (human embryonic lung fibroblasts MRC-5 cells	[94]
Rice albumin	DHHQ DAHK DHHK	Copper-induced LDL oxidation inhibition	[103]
Corn gluten	YFCLT	ABTS scavenging	[76]
	CSQAPLA YPKLAPNE YPQLLPNE	DPPH and O_2_^·‒^, ferric ions reducing capacity	[77]
	PF LPF	DPPH, ABTS scavenging	[48]
Corn protein	QQPQPW	DPPH, ABTS, O_2_^·ˉ^, HO^·^, reducing power, and iron chelating activity	[89]
	HALGA HAIGA AGLPM AGIPM	DPPH, ORAC, HO^·^, cellular antioxidant in HepG2 cells	[82]
Rye secalin	CQV QCV QVC QCA	Hydroxyl radical scavenging	[80]

Seven peptides derived from oat proteins were evaluated for ROO^·^ radical scavenging activities. Amongst them, GLVYI and YHNAP were the most potent with values of 0.67 and 0.61 μM TE/μM peptide, respectively while DVNNNANQLEPR had the least activity, 0.14 μM TE/μM [73]. Wheat-derived peptide, VLPPQQQY scavenging power against ABTS, DPPH, O_2_^·−^ and HO^·^ radicals were 371, 3977, 666 and 3629 mmol glutathione/mol peptide, respectively [74]. Rice bran protein hydrolyzed using trypsin followed by membrane and gel filtration chromatography separations afforded peptide YSK which exhibited high DPPH (IC_50_ 0.15 ± 0.01 mg/mL) [57]. Four other rice peptides showed DPPH (IC_50_ 0.144–0.161 mg/mL) and ABTS (EC_50_ 0.1070–0.658 mg/mL) scavenging activities [75]. An antioxidant peptide YFCLT, identified from corn gluten hydrolysate, exhibited excellent ABTS radical scavenging activity with EC_50_ value of 37.63 μM [76], while peptide CSQAPLA had IC_50_ values of 0.116 and 0.39 mg/mL in the DPPH and O_2_^·−^ tests [77].

#### 2.2.3 Radical scavenging mechanisms of cereal protein hydrolysates and peptides

There are two main mechanisms by which antioxidant molecules can deactivate free radicals: hemolytic or hydrogen atom transfer (HAT) and single electron transfer (SET). Both will produce identical end-products despite the difference in mechanisms [78]. The two mechanisms may occur in parallel, but one can dominate depending on the structure of the antioxidant peptide and the type of assay that will influence the solubility and partition coefficient.

**Fig. 1:**
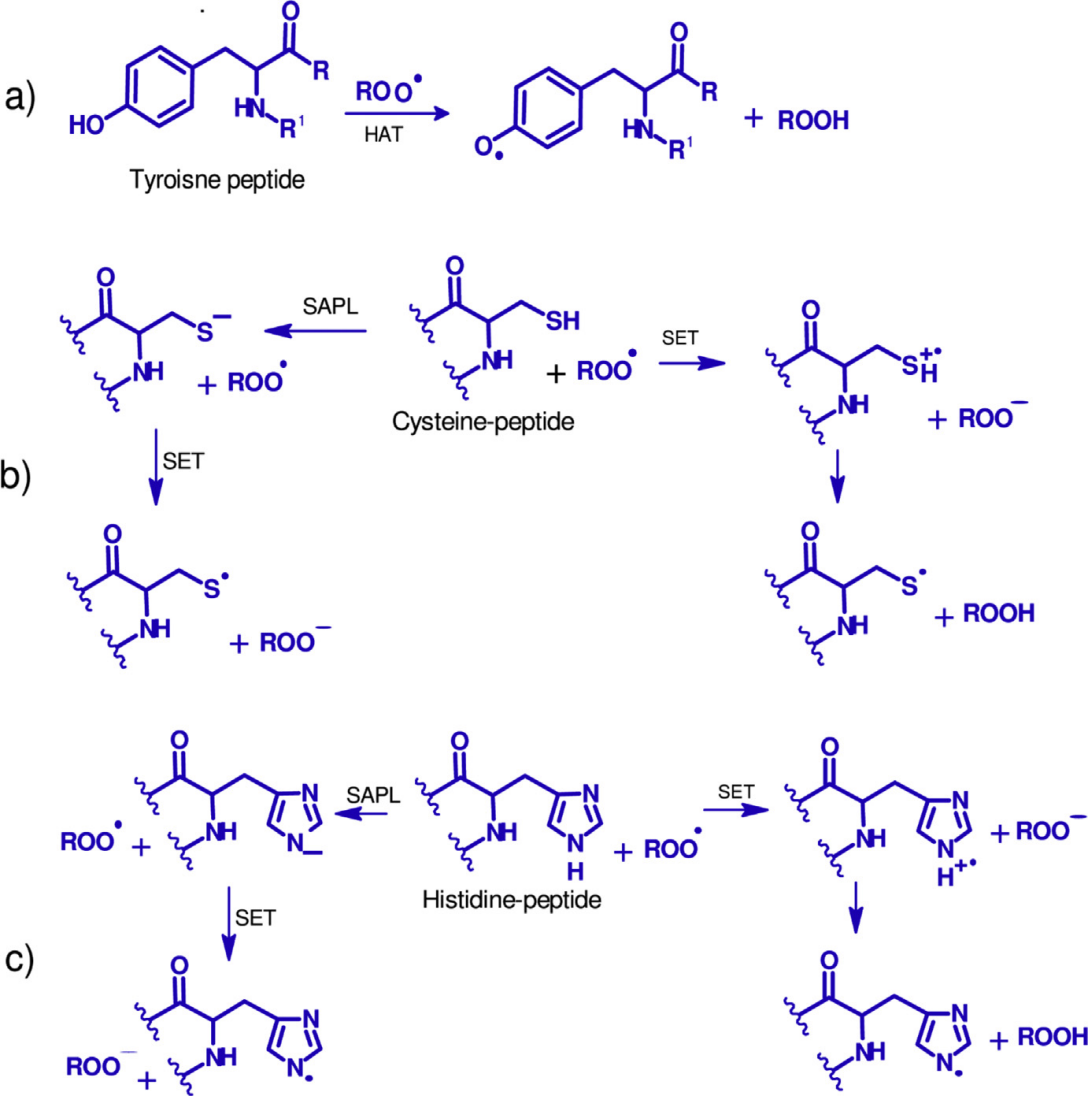
Radical scavenging mechanisms of peptides. HAT: hydrogen atom transfer, SET: single electron transfer, SAPL: solvent-assisted proton loss. Proposed scheme for tyrosine (a), cysteine (b) and histidine (c) containing peptides.

Tyrosine containing peptides can act mainly through a HAT mechanism while cysteine, tryptophan and histidine peptides act mainly via SET mechanisms. During HAT there is a hemolytic separation of the proton bound to the heteroatom as illustrated in Fig. 1.

The proton can be directly transferred between the reacting molecules, or can be involved in a solvent assisted proton loss (SAPL) depending on pH and acid-base properties [79]. Radicals of antioxidant peptides have a significantly longer life than hydroxyl and peroxyl radicals produced in foods and during oxidative stress. HAT-based methods like the oxygen radical absorbance capacity (ORAC) assay measure the ability of a molecule (e.g. peptide) to quench peroxyl radical (ROO^·^) by H-donation. It has been found that the reactivity is determined by the bond dissociation energy (BDE) of the H-X group in the antioxidant compound [80, 81]. Peptides that contain amino groups such as tyrosine, tryptophan, and histidine that can easily donate protons are, therefore, useful in the ORAC assay. A study on corn found that peptides HALGA and HAIGA have different activities in the ORAC assay, with the first one being about 1.6-fold more active than the second [82]. The two peptides have the same properties so one can assume that the replacement of leucine by isoleucine either caused a steric effect or reduced the ability of histidine to donate protons. Peptide GLVYIL from oats had higher ROO^·^ scavenging activity than YHNAP. It should be noted that the second peptide in addition to tyrosine, contains histidine, which is another proton donating amino acid [73]. The ROO^·^ activity of the oat peptides shows that the presence of proton donating amino acids and their actual location on the sequence are important. In the hydroxyl radical scavenging assay, the presence of cysteine and its location in tripeptides (CQV, QCV, QVC, QCA) derived from rye secalin was critical for their degree of activity. The mechanism occurred mainly through the homolytic cleavage of thiol (S—H) bonds as computed using density functional theory [80].

DPPH and ABTS assays are usually classified as SET reactions, meanwhile, these indicator radicals can be neutralized by reduction via electron transfers but also by quenching via the HAT mechanism [83]. The reactivity patterns of protein hydrolysates and peptide fractions are difficult to interpret because of the lack of detailed information about the composition and sequences of all peptides. The overall mechanism is also affected by the pH because bond dissociation energy and ionization potential of the reactive functional group are important [81, 84]. Ionization potential values generally decrease with increasing pH as does the electron donating capability. The sequential proton-loss electron-transfer (SPLET) mechanism, which involved the deprotonation of the antioxidant molecules followed by an electron transfer, was found to be important for DPPH radical scavenging activities of polyphenols [85, 86] and melatonin, a metabolite of tryptophan [87]. The method has not been applied to peptides likely because the knowledge of pKa values of an antioxidant molecule is crucial to assess the relative importance of the SPLET mechanism.

Residues of amino acids that are important for HAT mechanisms are also important for SET assays. In the absence of peptide sequences, the amount of some aromatic amino acids in hydrolysates can be used to determine the importance SET-based assays. For example, the tyrosine and phenylalanine content of rice bran hydrolysate and ultra-filtered fractions had positive correlations (*r* > 0.831) with their DPPH and ABTS radical scavenging activities [88]. In wheat, the presence of tryptophan or tyrosine at the C-terminus of peptides was important for their ABTS radical scavenging activities, however, for DPPH activities the location of these amino acids on sequences was less important [74]. In a related work, DPPH and ABTS scavenging activities of peptide QQPQPW from corn protein hydrolysate was due to the presence of tryptophan [89].

#### 2.2.4 Inhibition of lipid oxidation

Lipid molecules in foods are very prone to oxidation processes which is a major contributor to deterioration during manufacturing, storage, and distribution. Various models have been used to study how hydrolysed cereal proteins and peptides can prevent the oxidation of lipids through antioxidant mechanisms. Common systems include linoleic acid but also vegetable/animal fats, and food systems. Enzymatically generated wheat germ protein hydrolysates showed antioxidant activities in a linoleic system [90]. In another study, pepsin hydrolysed wheat germ proteins showed better oxidation of linoleic acid compared to hydrolysates generated by other proteases [66]. The fractionation of wheat gluten based on charge gave fractions that effectively inhibited lipid oxidation during cooking [47]. In a similar work, alcalase derived protein hydrolysates of rice residue prevented the oxidation of lard more efficiently than those from neutrase, bromelain and papain; the effect was partly attributed the presence of smaller size peptides [91]. It believed that hydrolyzed cereal proteins could increase meat shelf life since, for example, the incorporation of antioxidantrice peptide fractions into meat products decreased lipid oxidation by 19 and 15% after one and two weeks storage, respectively [92]. There is also a potential for use of cereal proteins in emulation because hydrolysis can convert them into very good functional and surface active molecules. In this regard, hydrolyzed rice proteins were shown to inhibit lipid oxidation in an oil-in-water emulsion [93]. In addition to hydrolysates, purified peptides have also been shown to prevent the oxidation of lipids. An antioxidant peptide isolated from hydrolyzed rice endosperm with the sequence FRDEHKK significantly inhibited the oxidation of linoleic acid [94]. The tetrapeptide DHHQ, and related DAHK and DHHK isolated from rice albumin showed antioxidant activities by inhibiting the oxidation of copper-induced oxidation of low-density lipoprotein (LDL).

#### 2.2.5 Mechanism of inhibition of lipid oxidation

**Fig. 2:**
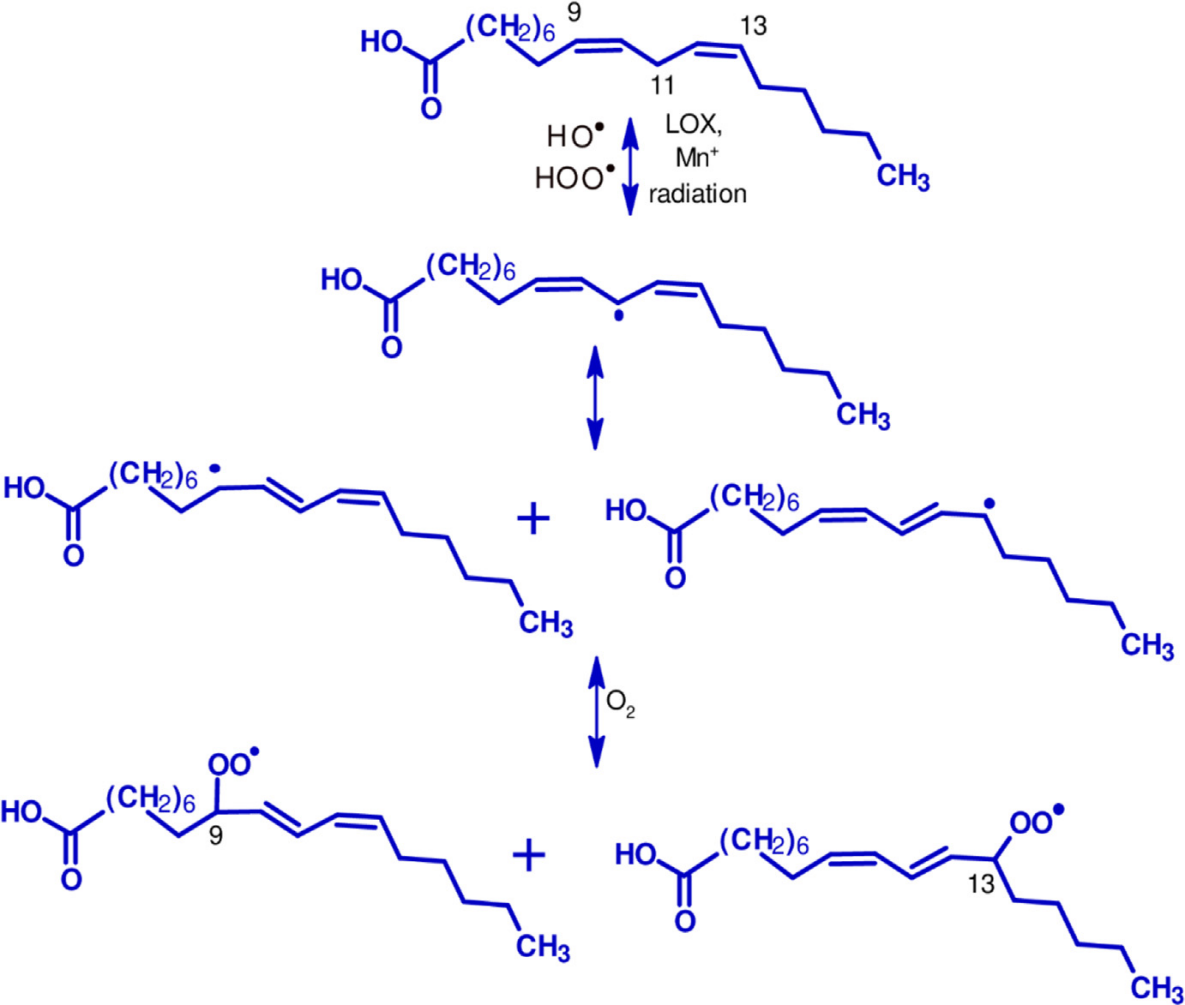
Formation of peroxyl radical from linoleic initiated by hydroxyl radical (HO^·^) hydroperoxide (HOO^·^), transition metal (Mn^+^), lipoxygenase (LOX), or radiation.

Oxidation of lipids in food and biological systems can be initiated by radical species but also by the presence of transition metals such as ferrous and copper ions. Transition metals are pro-oxidants and they decrease the oxidative stability of lipids through the decomposition of hydroperoxides into free radicals [95]. Metalloenzymes, especially lipoxygenases, are non-heme iron containing dioxygenases present in plant and mammals and can catalyze the oxidation of polyunsaturated fatty acids [96, 97]. The rate of initial oxidation is dependent on the reduction potential of the oxidant species which must be greater than that of the lipid. Hydrogen peroxide with reduction potential of 320 mV is, therefore, not able to directly initiate oxidation of unsaturated fatty acids (E ∼ 600 mV). Hydrogen peroxide does indirectly initiate lipid oxidation because it is the precursor for the formation of HO^·^ radicals, which is a strong initiator (E = 2300 mV) [98]. Thermodynamically, ROS with E > 1000 mV is capable of oxidizing polyunsaturated fatty acids [99]. The superoxide anion radical (O_2_^·−^, E = 940 mV) is, therefore, not strong enough to attract hydrogen from unsaturated fatty acids [100] while the hydroperoxy radical (HOO^·^, E = 1060 mV) can. Typical oxidative reactions of lipids are displayed in Fig. 2 using linoleic acid as a model.

**Fig. 3:**
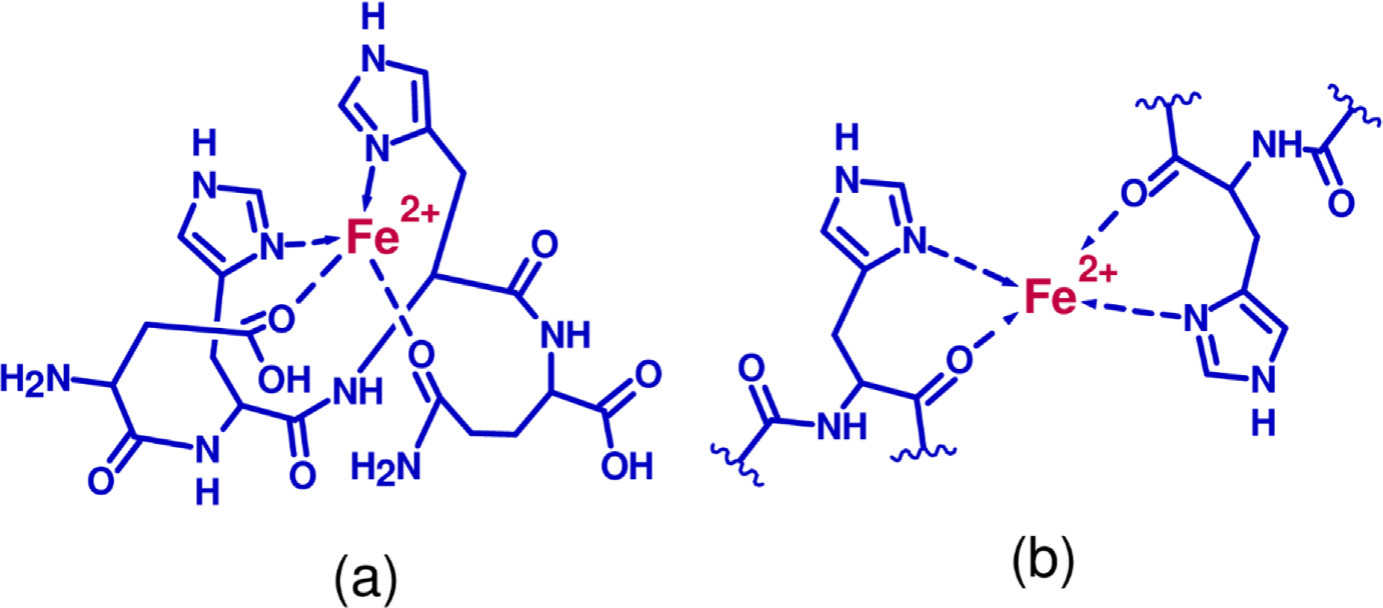
Proposed mechanism for the chelating of metal by peptide with multiple histidine. DHHQ (a) and FRDEHKK (b). residues like DHHQ (a) and peptides that contain a single histidine residue (b).

Hydrolyzed proteins and peptides like other antioxidant molecules can inhibit the oxidation of lipids in foods or biological systems through three established mechanisms. They can work through direct radical scavenging as described above and prevent proton abstraction from the lipid. Alternatively, they can donate protons to covert peroxyl radicals like those formed at C-9 and C-13 of linoleic acid (Fig. 2) to form less reactive hydroperoxides. These are the so-called chain-breaking antioxidants [101]. The other main mechanism is via metal chelation. The ability of hydrolyzed proteins from rice and corn to inhibit oxidation of lipids in meat, emulsions, and liposomes was believed to be due to their ability to chelate iron or copper ions, as well as their radical scavenging capacity [54, 92, 102]. Rice peptides FRDEHKK and DHHQ reduced the peroxidation of linoleic acid [94] and Cu^2+^-induced LDL oxidation mainly through their chelating mechanisms [103]. Both peptides contain histidine and aspartic acids which certainly contributed to the activity. The proposed intermediate states are displayed in Fig. 3. Two molecules are required for peptides with one chelating residue but not for those with multiple residues like DHHQ. It has been suggested that peptides with a histidine residue at the second or third position had high affinity for Cu^2+^ [103].

**Fig. 4:**
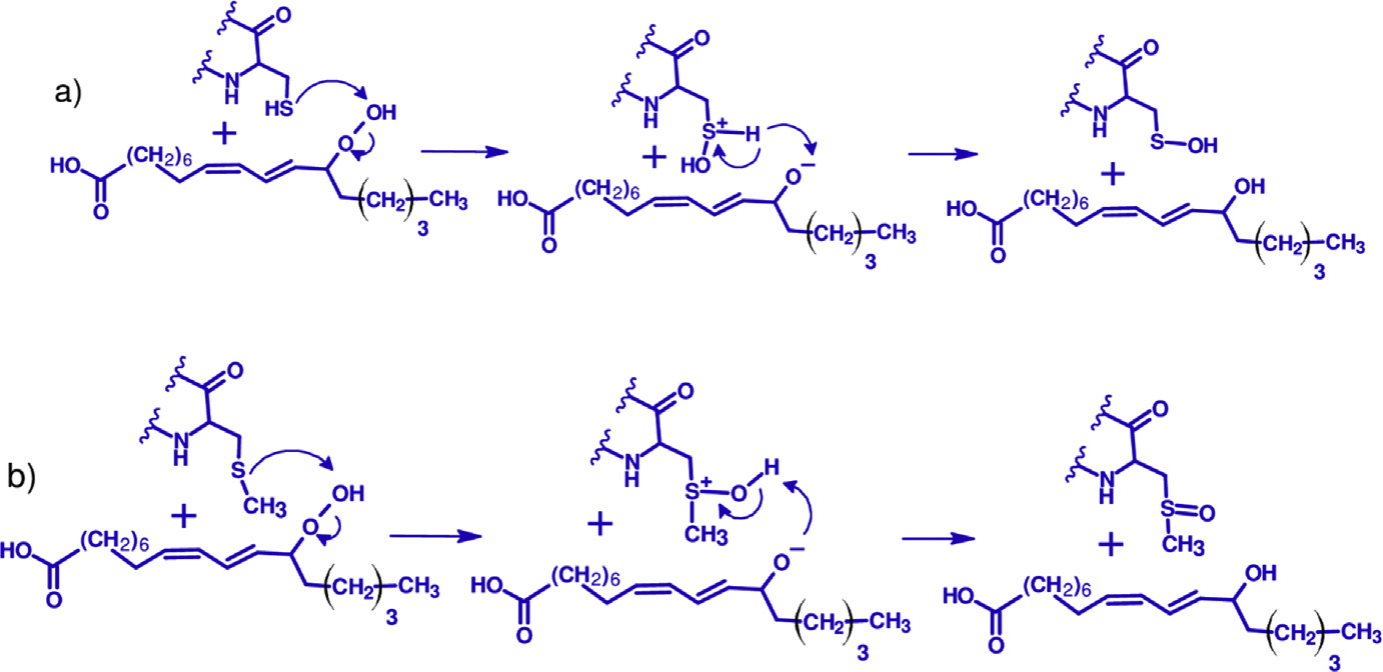
Proposed mechanism of the reduction of linoleic hydroperoxide by cysteine (a) or methionine (b) containing peptides.

Peptides can also reduce lipid hydroperoxides to relatively nonreactive lipid hydroxides through non-radical reactions. Gluten incubated with linoleic acid hydroperoxides and monolinolein hydroperoxides showed that most sulfhydryl groups were converted to a disulfide and other oxidation products [104]. The proposed reaction mechanism for the reduction of lipid hydroperoxides to lipid hydroxides can proceed through two-electron transfers from the sulfur of cysteine or methionine to yield sulfenic acid and sulfoxide derivatives (Fig. 4). In biological systems, enzymes such as peroxiredoxins or methionine sulfoxide reductases are able to reduce oxidized sulfur.

### 2.3 Antioxidant activity of cereal protein hydrolysates and peptides in cellular and animal models

Models other than in vitro chemistry and food-based models have been used to assess the antioxidant properties of cereal hydrolyzed proteins and peptides. In general, studies on cell and animal models are limited compared to in vitro tests. Rat adrenal gland pheochromocytoma (PC12) cells exposed to wheat germ protein alcalase hydrolysates maintained their integrity (i.e. no cytotoxicity) and, at 1 mg/ml, they prevented H_2_O_2_-induced oxidative stress by 63.7% [105]. In another study, wheat peptide fractions (2 mg/mL) prevented oxidative stress induced death in rat jejunal crypt (IEC-6) exposed to indomethacin and increased cell viability by 120% compared to the untreated cells [106]. Successive separation (<3 kDa) of rice bran hydrolyzed proteins by ion-exchange, size exclusion and RP-HPLC yielded seven antioxidant peptide fractions with anti-proliferative activity on human gastric cancer cells SGC-7901 [56]. Alcalase hydrolysates of proteins from brewers' spent grain prevented cell death and DNA damage human U937 monocytic blood cells exposed to hydrogen peroxide [107]. Only a few peptides have been investigated in cell and animal models. Peptide RVF from wheat had no cytotoxicity and protected human neuroblastoma cells (SH-SY5Y) from H_2_O_2_-induced cell death with an increase viability of 37% [108]. Peptide FRDEHKK from rice enhanced the viability of t-BOOH induced cytotoxicity in human MRC-5 fetal lung fibroblast cells (74%) and mouse RAW264.7 leukaemic monocyte macrophage (78%) [94]. Seven peptides derived from oats did not show any cytotoxicity at concentrations of 0–200 μM and four of them (FNDRLRQGQLL, GLVYIL, YHNAPGLVYIL, and DVNNNANQLEPR) protected HepG2 cells from peroxyl radical induced oxidative stress [73].

In rats treated with non-steroidal anti-inflammatory drugs, oxidative stress often increased and cause tissue damage in the small intestine. Daily intragastric administration of a low molecular weight wheat peptide fraction (140–1000 Da) for one month reduced edema and oxidative stress in the small intestine [109]. In a related work, the same wheat peptide fraction reduced ethanol-induced gastric mucosal damage in mice partly through an antioxidant mechanism [110]. Microscopic images showed that hepatocyte lesions induced in mice by Calmette-Guerin/lipopolysaccharide were reversed after supplementation of the diet with corn peptide fractions (600 mg/kg bodyweight) [111]. Low molecular weight (<5 kDa) peptide fraction from alcalase hydrolyzed corn proteins showed hepatoprotective effect against carbon tetrachloride -induced liver injury at 200 mg/kg body weight in mice [112]. Increased body fat is known to enhance oxidation of fatty acids that can be decreased by the addition of some antioxidant molecules to diets. In this regard, supplementation of high fat diets with antioxidant trypsin hydrolyzed oat proteins decreased oxidative stress in mice characterized by higher concentration of free thiol groups in plasma, higher activity of the superoxide dismutase antioxidant enzyme in plasma and liver as well as more vitamins E and C in liver [113].

#### 2.3.1 Mechanisms cereal protein hydrolysates and peptides in cellular and in vivo models

The mechanisms of antioxidant molecules at the cellular level can be through radical scavenging, regulation of the activity of redox enzymes or regulation and antioxidant response elements (AREs). Most ARES are genes activated by the nuclear factor erythroid 2 related factor 2 (Nrf2) and play a critical role in redox homeostasis, phase II metabolism, and cytoprotection during oxidative stress [114]. Both reactive oxygen species and reactive nitrogen species are present in vivo. Common antioxidant enzymes include superoxide dismutase (SOD), catalase (CAT), glutathione peroxidase (GPx), peroxiredoxins, glutaredoxin (Grx) and glutathione reductase [115]. Other enzymes such as aspartate aminotransferase (AST) and alanine aminotransferase (ALT) have been used to assess the oxidative status of the liver [111]. In the cytosol and mitochondria, SOD in the presence of copper zinc or manganese converts O_2_^·−^ radicals into H_2_O_2_ which is then converted in the peroxisome to water by CAT. In the cytoplasm and extracellular environments, GSHPx converts H_2_O_2_ and organic peroxides into water or alcohol derivatives while Prx acts on all peroxides and peroxynitrite (ONOO^−^) [115].

Most studied cellular mechanisms of hydrolyzed cereal proteins and peptides include regulation of antioxidant enzymes, available thiol groups, intracellular reactive oxygen species, and reduction of lipid oxidation. The hepatoprotective effect of a corn peptide fraction in mice was associated with a decrease in activities of amino acid transferases (AST, ALT) in serum while in the liver there was a decrease lipid oxidation and an increase in SOD activity and glutathione levels [112]. Wheat peptide fractions reduced ethanol-induced gastric mucosal damage in mice partly through an increased SOD, GPx, total antioxidant capacity level or decreased oxidation of lipid [110, 116]. The cytoprotective effect of wheat germ protein hydrolysate PC12 cells was explained by an increase in activity of CAT and SOD by 41.13% and 37.74%, respectively while its anti-apoptotic effect was due to the reduction (31.2%) of the fractional DNA content [105]. A related study explained the protection of stressed IEC-6 cells by small wheat peptides through an up-regulation of 11.1 and 8.6% of SOD and GPx, respectively [106]. The isolated wheat peptide RVF reduced apoptosis (6–14%) in SH-SY5Y cells and increased Bcl-2/Bax [108] proteins that regulate antioxidant pathway and cell death. In the case of brewers' spent grain, the protection was due to the reduction of DNA oxidation (human U937 monocytic cells) and the reduction of interferon-gamma (human Jurkat T cells), a proinflammatory cytokine [107]. The cytoprotection of HepG2 cells by oat peptides was attributed to several factors including the reduction of intracellular reactive oxygen species, increased cellular glutathione, and increased activities enzymes (SOD, CAT, GPx) [73].

## 3 Conclusions

Cereal peptides and hydrolyzed proteins have been shown to reduce oxidative stress in chemical-based assays, animal models, cell cultures, and food systems. Overall, only a small number of pure peptides have been investigated at a cellular level. There is, therefore, a need for more studies of diseases or conditions associated with oxidative stress in different types of cells and in animal models. There is a lack of human studies, even though peptides from digested food proteins are generally recognised as safe. Finally, more chemistry-based studies are also needed to investigate modifications that can occur to peptides due to temperature or high pressure.

## Declarations

### Author contribution statement

All authors listed have significantly contributed to the development and the writing of this article.

### Funding statement

This work was supported by the National Science and Engineering Research Council of Canada (Grant No: 371908).

### Competing interest statement

The authors declare no conflict of interest.

### Additional information

No additional information is available for this paper.
